# Large articulations do not increase wear rates of thin second-generation highly cross-linked polyethylene liners at ten years

**DOI:** 10.1302/2633-1462.411.BJO-2023-0124.R1

**Published:** 2023-11-06

**Authors:** Stuart A. Callary, Deepti K. Sharma, Taisha M. D’Apollonio, David G. Campbell

**Affiliations:** 1 Centre for Orthopaedic and Trauma Research, The University of Adelaide and Department of Orthopaedics and Trauma, Royal Adelaide Hospital, Adelaide, South Australia; 2 Centre for Orthopaedic and Trauma Research, The University of Adelaide, Adelaide, South Australia; 3 Wakefield Orthopaedic Clinic, Adelaide, Australia

**Keywords:** wear, radiostereometric analysis, total hip arthroplasty, implant design, cross-linked polyethylene, Bearing surface, highly cross-linked polyethylene, femoral heads, radiostereometric analysis (RSA), acetabular components, plain radiographs, acetabular liner, cementless THA, radiographs, hips, primary THA

## Abstract

**Aims:**

Radiostereometric analysis (RSA) is the most accurate radiological method to measure in vivo wear of highly cross-linked polyethylene (XLPE) acetabular components. We have previously reported very low wear rates for a sequentially irradiated and annealed X3 XLPE liner (Stryker Orthopaedics, USA) when used in conjunction with a 32 mm femoral heads at ten-year follow-up. Only two studies have reported the long-term wear rate of X3 liners used in conjunction with larger heads using plain radiographs which have poor sensitivity. The aim of this study was to measure the ten-year wear of thin X3 XLPE liners against larger 36 or 40 mm articulations with RSA.

**Methods:**

We prospectively reviewed 19 patients who underwent primary cementless THA with the XLPE acetabular liner (X3) and a 36 or 40 mm femoral head with a resultant liner thickness of at least 5.8 mm. RSA radiographs at one week, six months, and one, two, five, and ten years postoperatively and femoral head penetration within the acetabular component were measured with UmRSA software. Of the initial 19 patients, 12 were available at the ten-year time point.

**Results:**

The median proximal, 2D, and 3D wear rates calculated between one and ten years were all less than 0.005 mm/year, with no patient recording a proximal wear rate of more than 0.021 mm/year. Importantly, there was no increase in the wear rate between five and ten years.

**Conclusion:**

The very low wear rate of X3 XLPE liners with larger articulations remains encouraging for the future clinical performance of this material.

Cite this article: *Bone Jt Open* 2023;4(11):839–845.

## Introduction

The introduction of highly cross-linked polyethylene (XLPE) in total hip arthroplasty (THA) has resulted in a significant reduction of reported long-term wear rates compared to conventional polyethylene components.^1,2^ The Australian Orthopaedic Association National Joint Arthroplasty Registry (AOANJRR) has confirmed XLPE bearings to have significantly reduced revision rates at 16 years,^3^ which has led to the widespread adoption of XLPE components. A number of different manufacturing techniques are used to produce XLPE components including a different sequence of treatments including irradiation, annealing or melting of the polyethylene.^4^ One second-generation XLPE liner (X3; Stryker Orthopaedics, USA) is manufactured differently by sequentially irradiating and annealing the material to reduce in vivo oxidation.

While the majority of clinical studies report very low wear rates, large variations remain in the literature when investigating factors that may influence XLPE wear. For example, two retrospective studies of the X3 XLPE mid-term wear at five years reported a mean 2D wear rate of 0.106 mm/year and 0.109 mm/year,^5,6^ which is very close to the 0.100 mm/year threshold wear rate associated with osteolysis.^7^ However, the poor sensitivity of measurements made from plain radiographs in these retrospective studies is a significant limitation.^8^ XLPE has very low wear rates and requires more sensitive measures, such as radiostereometric analysis (RSA),^9^ to measure longer-term wear rates and investigate the effect of factors that may influence XLPE wear rates such as larger articulations.

Larger femoral heads are known to increase the stability of the hip joint by increasing the distance required for the femoral head to disengage from the acetabular component.^10,11^ A large multicentre randomized controlled trial found that patients who received a 36 mm articulation had significantly reduced incidence of dislocation (0.8%) when compared to patients who received 28 mm articulation (4.4%).^12^ Cohort studies have also confirmed a decreased risk of revision THA for dislocation or instability when larger articulations were used.^13-17^ RSA studies have confirmed that larger 36 mm articulations did not increase the wear rates of Longevity XLPE liners at three years^12^ or X3 XLPE liners at five years.^18^ However, to the best of the authors' knowledge, only one prospective RSA study has investigated the long-term wear of larger 36 mm articulations^19^ which was limited to only six hips available for analysis at long-term follow-up. The aim of this study was to measure the ten-year wear rate of thin X3 liners articulating with 36 and 40 mm articulations and compare these results to our previous RSA studies of 32 mm articulations with the same X3 XLPE liner at ten years.^20^

## Methods

This is a brief follow-up report of a prospective cohort previously reported at five years.^18^ The cohort originally included 19 patients who underwent primary THA with a 36 or 40 mm articulation between May 2007 and January 2009. All THAs in both cohorts were undertaken by the same surgeon (DGC) at Calvary Adelaide Hospital, Australia, and all patients received a second-generation XLPE liner (X3; Stryker Orthopaedics). Patients were eligible for inclusion if they had had a diagnosis of osteoarthritis; were aged between 45 and 80 years; were deemed suitable for an uncemented THA; and resided within the Adelaide metropolitan area. Ethical approval was obtained from the Institutional Ethics Committee and the trial is registered with Australian New Zealand Clinical Trial Registry (#ACTRN12616000952448). The X3 XLPE liner was manufactured using a cycle of 30kGy of γ irradiation followed by annealing at 130 °C for eight hours, repeated three times for a cumulative dose of 90kGy. All patients had flat liners within the same femoral stem with a cobalt chrome femoral head (Accolade; Stryker Orthopaedics). The need to use an acetabular component with an outer diameter less than 52 mm was an intraoperative exclusion criterion due to this resulting in a linear thickness below 5.8 mm with a 36 mm articulation.

### Ten-year follow-up

One patient was revised due to infection at five years; two patients were revised for taper wear at seven and eight years, respectively; one was revised for loose femoral stem at seven years; one died prior to ten year follow-up; and two patients were lost to follow-up leaving 12 hips to undergo RSA exams at ten years. Of the 12 patients examined at ten years, three required an acetabular component with an outer diameter of 54 mm received a 36 mm articulation with a resultant XLPE liner thickness of 5.9 mm; seven patients requiring an acetabular component with an outer diameter of 56 mm or 58 mm received a 40 mm articulation with a resultant minimum XLPE liner thickness of 5.8 mm; and two patients requiring an acetabular component with an outer diameter of 60 and 62 mm received a 40 mm articulation with a resultant minimum XLPE liner thickness of 7.4 mm (Table I). The results of this cohort will be compared to our recently published ten-year wear results of 16 patients who received a 32 mm articulation against the same X3 XLPE liner.^20^

**Table I.** Patient and implant details for the 32 mm and the larger 36/40 mm cohorts at ten years.

Cohort	32 mm	36/40 mm
Total, n	16	12
Sex, M:F	7:9	10:2
**Head size (mm), n**		
32	16	0
36	0	3
40	0	9
Side, L:R	13:3	2:10
**Cup outer diameter (mm), n**		
48	1	0
50	1	0
52	5	0
54	3	3
56	4	6
58	1	1
60	1	1
62	0	1
**Liner thickness (mm), n**		
5.8/5.9	2	10
7.4/7.9	8	2
9.9	5	0
11.4	1	0
Median age, yrs (range)	63 (47 to 73)	63 (55 to 76)
Median BMI, kg/m^2^ (range)	27.5 (22 to 30)	28 (22 to 35)
Inclination, degrees (range)	44 (39 to 58)	47 (40 to 50)

### RSA methodology

Supine RSA examinations were undertaken within the first postoperative week, at six months, and one, two, five, and ten years postoperatively. A ceiling-mounted radiological tube and a mobile radiological tube were used simultaneously to take exposures of the hip with a calibration cage (number 43; RSA Biomedical, Sweden). Penetration of the femoral head within the acetabular component was measured using UmRSA software (v6.0; RSA Biomedical). The outer ellipse and opening of the acetabular component were used to represent the acetabular segment for both cohorts. The maximum condition number and rigid body error accepted for each reference segment were 50 and 0.35 mm, respectively. Femoral head penetration was calculated in the medial, proximal and anterior axes. 2D head penetration was calculated as the vectorial sum of medial and proximal migrations and 3D head penetration was calculated as the vectorial sum of medial, proximal, and anterior migrations. The immediate postoperative radiographs provided a baseline for calculation of ‘bedding-in’ migration of the femoral head at one year.^21^ The slope of the penetration recorded for each individual between one and ten years was assumed to represent wear of the liner.

### Statistical analysis

The aim of this study was to determine whether the wear rate of larger 36 and 40 mm articulations was no greater than the wear rate of 32 mm articulations at ten years and a one-sided test of non-inferiority was considered the most appropriate statistical test. We previously reported that the median proximal wear rate between one and two years for the 32 mm cohort was 0.02 mm/year (interquartile range -0.04 to 0.07),^22^ and a non-inferiority margin of 0.03 mm was chosen (lower bound of the 90% confidence interval of the mean difference). An additional wear rate of 0.03 mm/year would result in a clinically relevant wear rate of 0.05 mm/yr, below which osteolysis is almost absent.^7^ Hence, the wear of the larger articulation cohort was not significantly greater than the 32 mm cohort if the non-inferiority was supported.

## Results

### Patient demographics

The median age of the 12 patients with larger articulations was 63 years (55 to 76) and the median BMI was 28 kg/m^2^ (22 to 35). The median cup size was 56 mm (54 to 62) with a median inclination of 47 (40 to 50). As evident, there was no difference in age and BMI between the 32 and 36/40 mm cohorts (Table I).

### Femoral head penetration

**Fig. 1:**
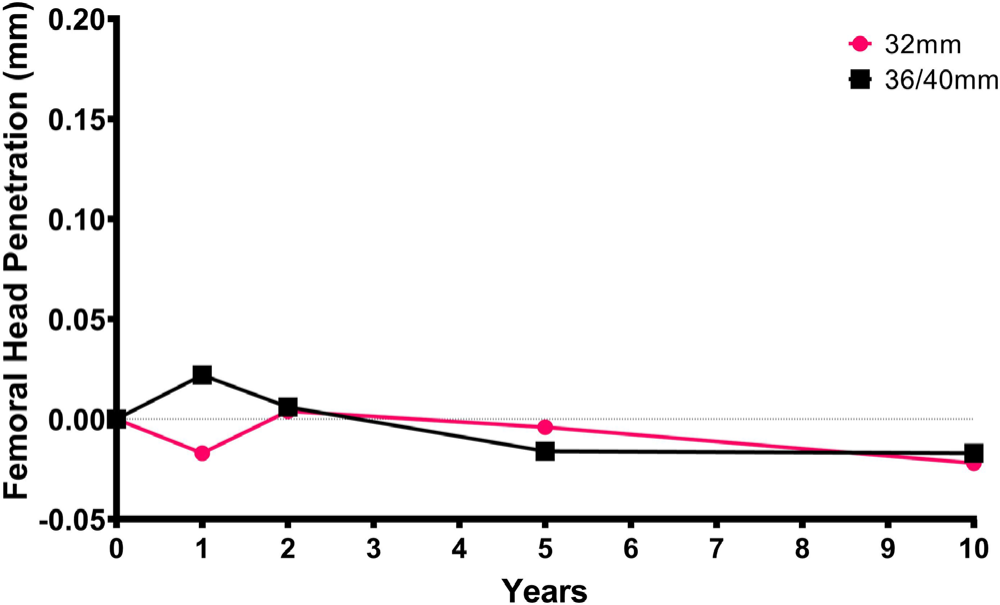
Median proximal femoral head penetration (mm) over time for the 32 and 36/40 mm cohorts.

**Fig. 2:**
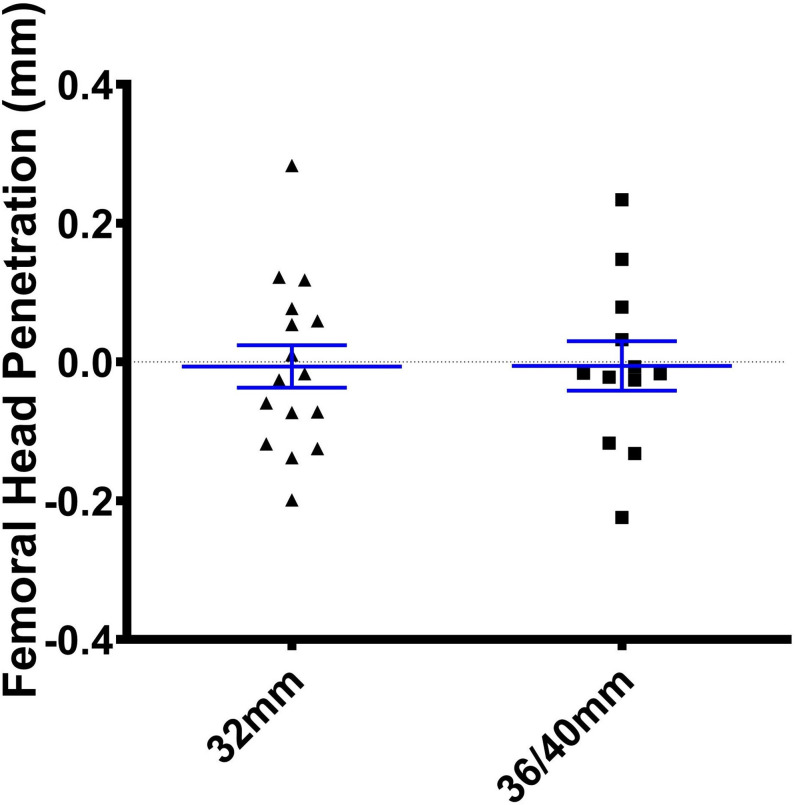
Proximal femoral head penetration (mm) between zero and ten years for the 32 and 36/40 mm cohorts.

The median medial, proximal, anterior, 2D,and 3D femoral head penetration between one week and ten years were –0.093,–0.017, 0.147, 0.163, and 0.278 mm, respectively (Table II). The majority of the head penetration occurred within the first year (Figure 1).^18^ The amount of head penetration in the proximal and medial directions was less than 0.3 mm at ten years for all hips in both cohorts (Figure 2).

**Table II.** Femoral head penetration, bedding-in, and wear for each cohort in each axis.

Variable		Medial (positive), lateral (negative)	Proximal (positive), distal (negative)	Anterior (positive), posterior (negative)	2D	3D
**Femoral head penetration between 1 wk and 10 yrs, mm**						
32 mm articulation	Median	-0.040	-0.022	-0.028	0.105	0.258
	Mean	-0.039	-0.007	-0.038	0.136	0.285
	SD	0.091	0.122	0.305	0.071	0.180
	Range	-0.186 to 0.134	-0.199 to 0.283	-0.837 to 0.369	0.069 to 0.313	0.0830 to 0.841
36/40 mm articulation	Median	-0.093	-0.017	0.147	0.163	0.278
	Mean	-0.079	-0.006	0.136	0.162	0.293
	SD	0.110	0.123	0.238	0.073	0.131
	Range	-0.283 to 0.086	-0.224 to 0.234	-0.255 to 0.552	0.042 to 0.294	0.150 to 0.580
	Difference between means	0.039	-0.001	-0.173	-0.025	-0.008
	90% lower CI limit of difference	-0.026	-0.081	-0.355	-0.072	-0.113
**Wear rate between 1 and 10 yrs, mm/yr**						
32 mm articulation	Median	-0.003	0.001	-0.018	0.002	0.004
	Mean	-0.000	0.003	-0.013	0.003	0.004
	SD	0.008	0.011	0.023	0.008	0.017
	Range	-0.010 to 0.018	-0.014 to 0.031	-0.042 to 0.037	-0.012 to 0.023	-0.026 to 0.042
36/40 mm articulation	Median	-0.011	-0.004	0.016	0.003	0.005
	Mean	-0.009	-0.001	0.011	0.006	0.011
	SD	0.009	0.014	0.025	0.010	0.016
	Range	-0.020 to 0.009	-0.027 to 0.022	-0.036 to 0.044	-0.007 to 0.021	-0.007 to 0.048
	Difference between means	0.009	0.004	-0.024	-0.004	0.006
	90% lower CI limit of difference	0.004	-0.004	-0.039	-0.010	-0.017
Non-inferiority supported		Yes	Yes	No	Yes	Yes
**Wear rate between 5 and 10 yrs, mm/yr**						
32 mm articulation	Median	-0.002	0.011	-0.013	0.005	0.007
	Mean	0.000	0.007	-0.021	0.003	0.002
	SD	0.014	0.016	0.037	0.018	0.028
	Range	-0.013 to 0.030	-0.013 to 0.043	-0.081 to 0.033	-0.025 to 0.048	-0.039 to 0.064
36/40 mm articulation	Median	-0.007	0.002	0.036	0.011	0.033
	Mean	-0.012	0.004	0.016	0.013	0.030
	SD	0.017	0.010	0.050	0.015	0.031
	Range	-0.048 to 0.012	-0.009 to 0.021	-0.063 to 0.078	-0.003 to 0.048	-0.046 to 0.083
	Difference between means	0.012	0.003	-0.037	-0.010	-0.028
	90% lower CI limit of difference	0.002	-0.006	-0.065	-0.021	-0.047
Non-inferiority supported		Yes	Yes	No	Yes	No

### Wear rate

**Fig. 3:**
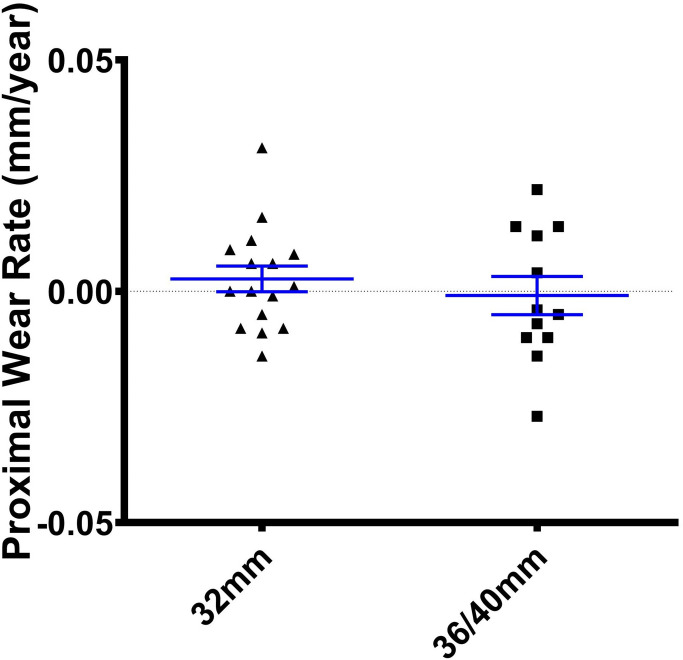
Proximal wear rate (mm/year) between one and ten years for the 32 and 36/40 mm cohorts.

The median medial, proximal, anterior, 2D, and 3D wear rate for larger articulations between one and ten years were -0.011,–0.004, 0.016, 0.003, and 0.005 mm/year, respectively (Table II). The median medial, proximal, anterior, 2D, and 3D wear rates for the 32 mm articulations at ten years were -0.003, 0.001, 0.002 and 0.004 mm/year, respectively. Non-inferiority of medial, proximal, 2D, and 3D wear of the larger articulations was supported when compared to 32 mm articulations. No patient in either of the 32 or 36/40 mm cohorts had a medial, proximal, or 2D wear rate greater than 0.05 mm/year. The maximum proximal wear rate was 0.031 and 0.022 mm/year for the 32 and 36/40 mm cohorts respectively (Figure 3). Importantly, the wear rate in each axis between five and ten years was similar to that between one and five years. The median medial, proximal, anterior, 2D, and 3D wear rates for larger articulations between five and ten years were -0.007, 0.002, 0.036, 0.011, and 0.033 mm/year, respectively.

## Discussion

The introduction of XLPE in THA has significantly reduced polyethylene wear.^1^ This reduction in wear has encouraged the use of larger articulations, which have been shown to reduce the rates of early dislocation.^12,17^ Implant Registry data have reported widespread acceptance of XLPE, improved clinical results and increased use of femoral heads 32 mm or larger in primary THA.^23^ However, longer-term effects of larger articulations with relatively thin XLPE liners on in vivo wear are limited. Our study found negligible wear of larger articulations at ten years similar to that previously reported for 32 mm articulations against the same XLPE liner.

There are only two long term clinical studies of X3 XLPE liners used with larger articulations (Table III).^24,25^ Remily et al^24^ reported a combined mean 2D wear rate of 0.015 mm/year at 11 years (10 to 14 years) for 101 primary THA patients treated with metal or ceramic head sizes of 28, 32, 36 and 40 mm. Sax et al^25^ reported a mean 2D wear rate at ten years (8 to 15) for 69 primary THA patients of 0.02 mm/year for 36/40 mm articulations, which was no greater than 0.02 mm/year for 28/32 mm articulations (p = 0.074). The 2D wear rate in our study is 80% and 85% lower than Remily et al^24^ and Sax et al,^25^ respectively. However, both these studies used Martell Hip Analysis software to measure the wear rates retrospectively on plain radiographs which have reduced sensitivity and precision. Similarly, Deckard et al^5^ and Selvaraj et al^6^ reported a much higher 2D wear rate between one and five years of 0.106 and 0.109 mm/year for 36 mm articulations against X3 XLPE liners.^6^ If these wear rates were true, it would imply that a high percentage of these hips were at risk of developing osteolysis,^6,7^ and would be of concern for the surgeons using X3 XLPE liners with large articulations. Careful interpretation of the method used and an understanding of RSA methodology as a gold standard technique to measure XLPE wear rates is required.^9,21^

**Table III.** Long-term ( ≥ ten years) wear studies of X3 highly cross-linked polyethylene acetabular components.

Wear study	Cohort no. analyzed	Head size, mm	Follow-up, yrs (range)	Wear rate calculation	Method	Mean proximal wear rate, mm/yr (SD)	Mean 2D wear rate, mm/yr (SD)	Mean 3D wear rate, mm/yr (SD)
Remily et al, 2021^24^	101	28 to 40 Ceramic and CoCr	11.1 (10.4 to 14.4)	0 to 10*	Martell Hip Analysis	N/R	0.015 (0.018)	N/R
Sax et al, 2022^25^	51	28 to 32 Ceramic and CoCr	10 (8 to 15)	0 to 10*	Martell Hip Analysis	N/R	0.02	N/R
	69	36 to 40 Ceramic and CoCr	10 (8 to 15)	0 to 10*	Martell Hip Analysis	N/R	0.02	N/R
Campbell et al, 2022^20^	16	32 CoCr	10 (8 to 15)	1 to 10	RSA	0.004 (-0.004 to 0.009)†	0.000 (-0.005 to 0.005)†	0.002 (-0.004 to 0.004)†
Current study	12	36 + 40 CoCr	10	1 to 10	RSA	-0.004 (0.014)	0.003 (0.010)	0.005 (0.016)

* Initial postoperative radiograph used described as within first year.

† Median (interquartile range).

N/R, not reported; RSA, radiostereometric analysis; SD, standard deviation.

Besides the two cohorts described in this study, there are no other long-term (≥ ten years) prospective RSA studies of the X3 XLPE liners (Table IV). We have previously reported wear rate for X3 liner is negligible when coupled with 32 mm articulations.^20^ In the current RSA study, we report for the first time a wear rate below 0.005 mm/year between one and ten years for X3 XLPE liner with larger articulations. Similar very low median proximal wear rates below 0.010 mm/year were reported for other XLPE liners at ten years.^1,19,26,27^ Only one RSA study of larger articulations reported a median proximal wear rate of 0.002 mm/year for 36 mm articulations,^19^ which is similar to our findings. One long-term RSA study did report a proximal wear rate of 0.020 mm/year,^28^ but did not exclude bedding-in between three months and one year. The exclusion of bedding-in within the first year is known to be an important methodological consideration,^21^ as more recently highlighted by Khosbin et al.^2^ Bedding-in likely contributed to considerably larger 3D wear rates of 0.066,^29^ 0.067,^30^ 0.055,^31^ and 0.042mm/year, respectively,^32^ being reported in long-term studies of XLPE liners that used a unique retrospective RSA methodology with no baseline examination.

**Table IV.** Prospective long-term ( ≥ ten years) radiostereometric analysis wear studies of highly cross-linked polyethylene acetabular components.

RSA study	XLPE implant	Head size, mm	Head material	Cohort no. analyzed	Follow-up, yrs	Mean proximal wear rate, mm/yr (SD)	Mean 3D wear rate, mm/yr (SD)
Rohrl et al, 2012^26^	Osteonics Cup (Crossfire PE; Stryker)	28	CoCr	8	10	0.002*	N/R
Johanson et al, 2012^27^	XLPE Cup (Durasul; Zimmer)	28	CoCr	23	10	0.005 (SE 0.002)*	0.005 (SE 0.002)*
Glyn-Jones et al, 2015^1^	Longevity XLPE liner (Trilogy Cup; Zimmer)	28	CoCr	20	10	0.009 (0.066)	0.003 (0.023)
Nebergall et al, 2016^19^	Longevity XLPE liner (Trilogy Cup; Zimmer)	28	CoCr	6	13	0.007 (SE 0.003)	N/R
		36	CoCr	6	13	0.002 (SE 0.003)	N/R
Bergvinsson et al, 2021^28^	HXLPE liner (CSF Plus Cup; JRI Orthopaedics)	28	CoCr	15	10	0.020†	0.020†
Campbell et al, 2022^20^	X3 XLPE liner (Trident Cup; Stryker)	32	CoCr	16	10	0.004 (-0.004 to 0.009)‡	0.002 (-0.004 to 0.004)‡
Current study	X3 XLPE liner (Trident Cup; Stryker)	36 + 40	CoCr	12	10	-0.004 (0.014)	0.005 (0.016)

* Calculated between two years and ten years.

† Calculated between three months and ten years.

‡ Median (interquartile range).

N/R, not reported; SD, standard deviation; SE, standard error.

A strength of this study is the use of a similar historical control cohort of smaller 32 mm heads. While not part of the same study, introduction of the larger heads (≥ 36 mm) following confirmation of the low wear rates of 32 mm heads against the new X3 liner adheres to the stepwise introduction of new implant designs advocated in orthopaedics.^33,34^ The main limitation of our study is a small sample size which is common to most long-term RSA studies. However, a post hoc power analysis confirmed the adequacy of the sample sizes used in the comparison at ten years. Overall, 12 patients (six in each cohort) are required to be 95% sure that the lower limit of a one-sided 97.5% confidence interval (or equivalently a 95% two-sided confidence interval) will be above the non-inferiority limit of -0.03. A second limitation is the use of the outer ellipse as the acetabular reference segment in our RSA results presented for both cohorts. This accounts for very small differences in the ten-year RSA results of the 32 mm articulation control group previously reported,^20^ which used a combination of the tantalum markers placed in the outer rim of the liner and the ellipse of the metal shell as the acetabular reference segment. Ellipse reference segments have a similar accuracy to those that include markers in the rim,^9^ but the combined method has been shown to have the superior precision.^35^ Third, an important limitation of this wear study was the inability to investigate non-wear-related consequences of larger diameter femoral heads, such as the potential for increased corrosion and metal release at the head-neck taper junction of larger articulations.^36^ In this small series, two hips were revised at seven and eight years due to taper corrosion.^37^

In conclusion, larger articulations did not significantly increase the proximal, medial, 2D, or 3D wear rates between one and ten years of the X3 XLPE liner when compared to our previous study of 32 mm articulations. These results are promising for the continued use of larger articulations, particularly in those patients, such as the elderly, in whom a decreased risk of dislocation is a priority.

**Take home message**

- There is negligible wear of highly cross-linked polyethylene (XLPE) liners articulating with large femoral head sizes after ten years.

- The very low long-term wear rate of X3 XLPE liners is encouraging for the future performance of this implant.
